# Conduction System Pacing versus Conventional Biventricular Pacing for Cardiac Resynchronization Therapy: Where Are We Heading?

**DOI:** 10.3390/jcm12196288

**Published:** 2023-09-29

**Authors:** Giulia Domenichini, Mathieu Le Bloa, Cheryl Teres Castillo, Denis Graf, Patrice Carroz, Ciro Ascione, Alessandra Pia Porretta, Patrizio Pascale, Etienne Pruvot

**Affiliations:** Cardiology Service, University Hospital of Lausanne, Rue du Bugnon 46, 1011 Lausanne, Switzerland

**Keywords:** cardiac resynchronization therapy, conduction system pacing, biventricular pacing

## Abstract

Over the last few years, pacing of the conduction system (CSP) has emerged as the new standard pacing modality for bradycardia indications, allowing a more physiological ventricular activation compared to conventional right ventricular pacing. CSP has also emerged as an alternative modality to conventional biventricular pacing for the delivery of cardiac resynchronization therapy (CRT) in heart failure patients. However, if the initial clinical data seem to support this new physiological-based approach to CRT, the lack of large randomized studies confirming these preliminary results prevents CSP from being used routinely in clinical practice. Furthermore, concerns are still present regarding the long-term performance of pacing leads when employed for CSP, as well as their extractability. In this review article, we provide the state-of-the-art of CSP as an alternative to biventricular pacing for CRT delivery in heart failure patients. In particular, we describe the physiological concepts supporting this approach and we discuss the future perspectives of CSP in this context according to the implant techniques (His bundle pacing and left bundle branch area pacing) and the clinical data published so far.

## 1. Introduction

Conduction system pacing (CSP) using His bundle or left bundle branch stimulation has been associated with better clinical outcomes compared to conventional right ventricular pacing in patients implanted for bradycardia indications [1,2]. Indeed, stimulation at the conduction system level provides a more physiological ventricular activation and reduces the acute and long-term effects related to dyssynchronous right ventricular pacing [3]. Because of this clinical evidence and the improvements in implanting techniques, CSP has become the new standard approach for conventional bradycardia indications and is gaining consensus as an alternative or in addition to conventional biventricular (BiV) pacing to deliver cardiac resynchronization therapy (CRT). However, despite promising preliminary data [4,5,6,7,8,9,10,11,12,13,14,15,16], the lack of large randomized controlled trials prevents CSP from being used routinely in clinical practice for CRT delivery.

This review article provides a state-of-the-art of CSP “*in lieu*” of conventional biventricular pacing in heart failure patients. In particular, we describe the physiological concepts supporting the rationale of CSP and the different CSP approaches to delivering CRT. Furthermore, we discuss the results of the main clinical studies published so far and how to translate this evidence into clinical practice to identify the optimal approach for CRT delivery according to the patient’s characteristics on an individual basis.

## 2. Conduction System Pacing to Deliver Cardiac Resynchronization Therapy

### 2.1. Rational and Techniques

The attempts to improve electro-mechanical synchronization in heart failure patients with wide QRS complexes have historically been based on biventricular stimulation delivered by a conventional endocardial right ventricular lead and an additional lead implanted on the epicardial left ventricular surface via the coronary venous system [17,18]. However, pacing from the epicardium to the endocardium is not physiological and makes restoring electrical ventricular synchronicity challenging, especially in patients with non-left bundle branch block (LBBB) patterns and a relatively narrow QRS duration, despite targeting the latest electro-mechanical implantation site for the left ventricular lead and programming optimized atrioventricular and interventricular intervals in CRT devices.

The attractiveness of CSP is based on the concept that recruiting the intrinsic conduction pathways at the His bundle or at the left bundle branch level would allow a more physiological activation of cardiac myocytes, leading to better mechanical synchronicity [19]. In acute hemodynamic studies on unselected populations with standard CRT indications, CSP delivered in the His bundle region [4,20] or in the left bundle branch area [20,21] is associated with better left ventricular and biventricular synchronicity and hemodynamics compared to biventricular pacing by significantly reducing left ventricular and biventricular activation time and biventricular dyssynchrony index [21]. However, these results seem to be attenuated by the presence of a septal scar [21], and left bundle branch area pacing (LBBAP) may induce a delay in right ventricular activation compared to His pacing [20].

The initial experiences using CSP for CRT delivery have been performed by pacing the His bundle region. In the randomized *His-Sync Pilot Trial* [5], the His-CRT patients showed a greater QRS narrowing and a trend towards a higher improvement in left ventricular ejection fraction (LVEF) compared to the BiV-CRT patients, but the study was limited by the high rates of crossover in the BiV-CRT group, mainly because of the inability to correct the QRS owing to nonspecific intraventricular conduction delays. The *His-Alternative Trial* [22] randomized patients with left bundle branch block and CRT indications to His vs. biventricular pacing. His corrective pacing was achieved in 72% of the His-CRT patients. At six-month follow-up, a similar increase in LVEF was observed in both groups, but significantly higher pacing thresholds were documented in the His-CRT group. These preliminary results on His pacing highlighted the concepts that patients with advanced cardiomyopathy often present multiple electrical dyssynchronies to be treated and that electrical resynchronization can be more effective by combining stimulation from the specialized conduction system with conventional epicardial left ventricular stimulation able to recruit myocardial areas with late electrical activation. In a feasibility study from Vijayaraman et al. [8], a combined His-LV stimulation approach (His-Optimized CRT (*HOT-CRT*)) was associated with a significant QRS narrowing compared to either His pacing or BiV pacing alone and a significant improvement in LVEF at mid-term follow-up. However, because of the observational nature of the study, the applicability of this approach in routine clinical practice remains to be validated.

Despite preliminary encouraging results, technical difficulties in achieving the target pacing site, unsatisfactory electrical lead parameters especially in terms of increase in pacing thresholds over time [23,24], and the inability to correct infra-Hisian or more distal conduction diseases [25] limit the adoption of His pacing as the technique of choice to deliver standard bradycardia pacing or as an alternative to conventional biventricular pacing. 

Over the last few years, a new approach for CSP has been developed consisting of pacing the left bundle branch area to recruit directly the pre-divisional portion of the left bundle branch (LBB pacing “*sensu stricto*”) or, more distally, the left fascicular branches. The left septal pacing is considered a part of the LBBAP, though it does not directly activate the left conduction system [26]. Compared to His pacing, the advantages of LBBAP are the possibility to correct infra-Hisian blocks, the stability of the pacing parameters over time [2,27], and the higher implant success rate [28,29]. For all these reasons and the encouraging results obtained in bradycardia indications [2], LBBAP has been rapidly introduced as a promising alternative to His pacing for CRT delivery. In a cohort of 325 patients with LVEF < 50% and CRT indication, LBBAP was successfully obtained in 85% of patients and associated with a significant reduction in QRS duration (from 152 ± 32 to 137 ± 22 ms, *p* < 0.01) and an improvement in LVEF at 6-month follow-up (33 ± 10% to 44 ± 11%, *p* < 0.01) [6]. In this study, the presence of an LBBB was an independent predictor of echocardiographic response. However, as documented in a subsequent series from the same study group [7], also patients showing a right bundle branch block (RBBB) pattern may benefit from LBBAP and the mechanism leading to QRS narrowing in these specific cases owes to a combination of non-selective LBB capture [30].

In terms of safety, the *MELOS Study* [29], the largest observational registry on LBBAP outcomes, describes an overall complication rate of 11.7%, including acute and late complications, which is comparable with the data previously reported for BiV-CRT implantations [31]. In particular, a total of 8.3% of complications were related to the LBBAP lead, including 3.7% of acute left ventricular perforations, managed by lead repositioning and not associated with adverse clinical consequences, and 1.5% of lead dislodgements. More recently, cases of interventricular septal hematoma have also been reported as a complication of LBBAP lead implantation [32,33,34]. All these observations highlight the fact that refinements in the LBBAP delivery systems and dedicated LBBAP leads would substantially contribute to the reduction in the implantation complication rate and hopefully they will be available in the near future.

The adjunct of conventional epicardial left ventricular stimulation to LBBAP has also been proposed, like for the His-bundle pacing, to overcome the inability to correct distal conduction disease in the His–Purkinje system or inside the myocardium. In a feasibility study from Jastrzębski et al. [9], LBBAP-optimized CRT (*LOT-CRT*) was attempted in 112 consecutive patients with standard CRT indications and eventually obtained in 81%. LOT-CRT was associated with greater electrical resynchronization in terms of QRS narrowing compared to BiV-CRT and LBBAP alone, as well as to a significant improvement in the echocardiographic and clinical parameters at 3-month follow-up compared to baseline. In this series, the complications rate was relatively low, accounting for five cases of early complications (one LBBAP and one coronary sinus lead displacement; one septal perforation with LBBAP lead; two pocket hematomas) and three complications that occurred during follow-up (one infection; one increase in coronary sinus lead pacing threshold; one right atrial lead dislodgement). Based on these preliminary results, LOT-CRT can be considered an alternative to conventional biventricular pacing in cases of suboptimal electrical resynchronization. However, larger randomized studies are still needed to support its use in routine clinical practice.

**Figure 1:**
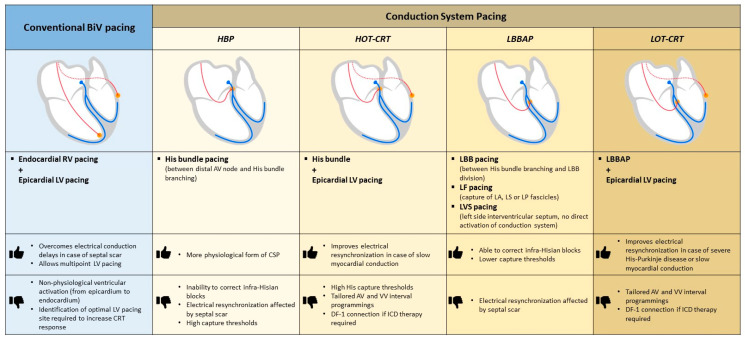
CRT implant techniques (standard biventricular pacing and conduction system pacing): anatomical differences, advantages, and limitations. Abbreviations: AV: atrioventricular; BiV: biventricular; CRT: cardiac resynchronization therapy; CSP: conduction system pacing; HBP: His bundle pacing; HOT-CRT: His optimized-cardiac resynchronization therapy; ICD: implantable cardioverter defibrillator; LA: left anterior; LBB: left bundle branch; LBBAP: left bundle branch area pacing; LF: left fascicular; LS: left mid-septal; LOT-CRT: left bundle branch area pacing optimized-cardiac resynchronization therapy; LP: left posterior; LV: left ventricular; LVS: left ventricular septal; RV: right ventricular; VV: interventricular.

Figure 1 illustrates the anatomical differences, advantages, and limitations of standard biventricular pacing and conduction system pacing implant techniques.

Table 1 summarizes the results from the largest series evaluating the feasibility and clinical outcomes of CSP techniques for CRT delivery.

### 2.2. LBBAP: Comparison with Biventricular Pacing and Implications in Clinical Practice

Data from relatively large observational studies [10,13,15] and small randomized series [12,14] comparing CSP to conventional BiV pacing confirmed the preliminary observations attesting to the safety and clinical benefits of CSP for CRT delivery. In a retrospective series of 477 patients undergoing CRT, the primary outcome of death or heart failure hospitalization was significantly lower in the CSP group (including His bundle pacing (*n* = 87) and LBBAP (*n* = 171)) compared to the BiV group (28.3% vs. 38.4%; HR 1.52; 95% CI 1.082–2.087; *p* = 0.013) after a mean follow-up of 27 ± 12 months, and the extent of these results was more prominent in patients showing an LBBB pattern at baseline [10]. Similar results have been recently documented in a larger retrospective study of 1778 CRT patients where LBBAP alone was compared to BiV CRT, showing a significant reduction in death or heart failure hospitalization (20.8% vs. 28%; HR-1.495; CI 1.213–1.842; *p* < 0.001) after a mean follow-up of 33 ± 16 months [13]. Importantly, the incidence of procedural complications in this series was significantly higher in the BiV group compared to the LBBAP group (7.5% vs. 3.8%, *p* < 0.001). The LBBP has also been associated with a significant improvement in LVEF at 6-month follow-up (mean difference: 5.6%; 95% CI: 0.3–10.9; *p* = 0.039) in the *LBBP-RESYNC Trial* [12], where 40 patients with nonischemic cardiomyopathy and CRT indications were randomized to LBBP-CRT vs. BiV-CRT.

According to these results, CSP could be considered an alternative to conventional biventricular pacing for CRT candidates, especially when delivered as LBBAP, but the lack of large randomized studies precludes spreading this approach in daily practice. Indeed, according to the current guidelines [31,35,36], CSP may be offered only as a bail-out option in CRT patients in whom coronary sinus lead implantation is unsuccessful or as an alternative to standard BiV to maintain physiological ventricular activation in patients with mild left ventricular dysfunction and expected to require a high burden of ventricular pacing, or in patients with tachycardia-induced cardiomyopathy in the context of an “ablate and pace” strategy. Therefore, to translate these recommendations into clinical practice, CSP could be reasonably offered to elderly patients with low LVEF, several comorbidities, and bradycardia indications for ventricular pacing as an alternative to conventional BiV pacing, with the aim to reduce the complication rates related to conventional coronary sinus lead implantation and the costs of sophisticated CRT devices, but also to CRT non-responders or CRT candidates with non-LBBB patterns where there is evidence of a relatively proximal His–Purkinje conduction disease.

**Figure 2:**
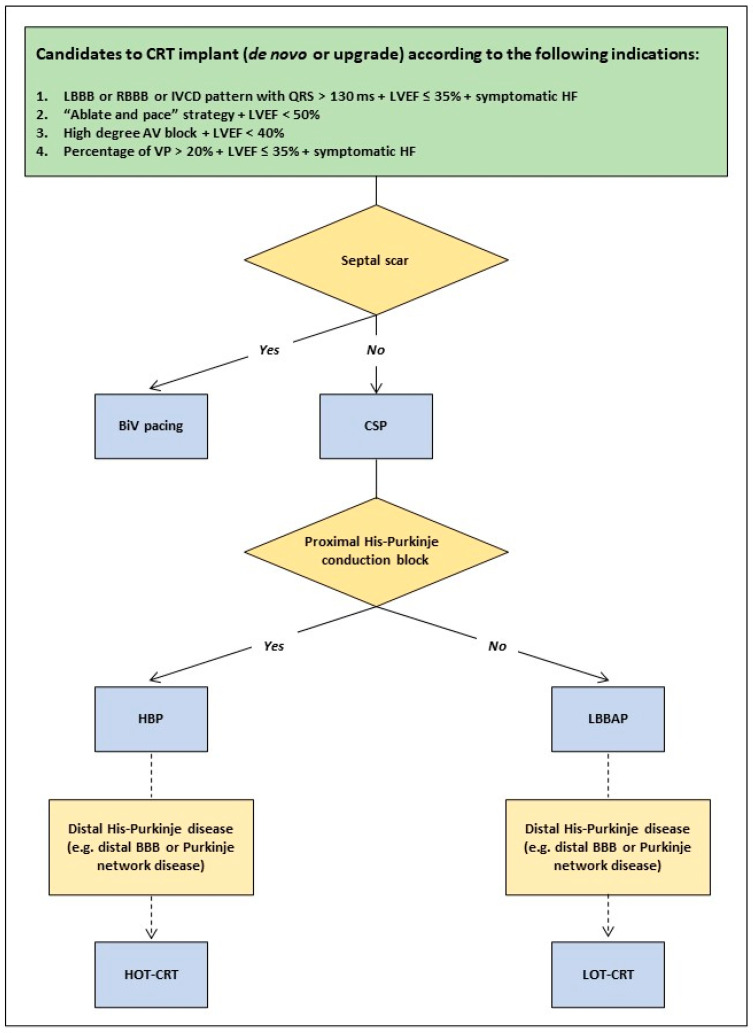
Decision tree to guide the strategy for cardiac resynchronization therapy (CRT) implants in heart failure patients according to the patient’s clinical characteristics and the different implant techniques. Abbreviations: BBB: bundle branch block; BiV: biventricular; CSP: conduction system pacing; HF: heart failure; HBP: His bundle pacing; HOT-CRT: His optimized-cardiac resynchronization therapy; IVCD: intraventricular conduction delay; LBBAP: left bundle branch area pacing; LBBB: left bundle branch block; LVEF: left ventricular ejection fraction; LOT-CRT: left bundle branch area pacing optimized-cardiac resynchronization therapy; RBBB: right bundle branch block; VP: ventricular pacing.

Indeed, in a mechanistic study with simulated ventricular activation on 24 4-chamber heart geometries, including His–Purkinje systems with proximal LBBB, Strocchi et al. [37] documented that septal scar and severe left ventricular His–Purkinje conduction disease attenuate the benefits of CSP, whereas BiV stimulation alone, in case of septal scar, or in addition to CSP as HOT-CRT or LOT-CRT, in case of severe left ventricular His–Purkinje conduction disease, would significantly improve biventricular activation time. These experimental results support the importance of maintaining conventional epicardial left ventricular stimulation as an option in specific conditions and highlight the concept of an individualized approach for CRT delivery based on the underlying electrical and myocardial disease. Based on these observations provided by Strocchi et al. [37] and the evidence from the clinical studies published so far [5,6,7,8,9,22,25], we might tentatively propose a decision tree for CRT delivery in heart failure patients who are CRT candidates according to the current recommendations [31,35], which is shown in Figure 2. In particular, BiV pacing should be adopted in cases of septal scars to overcome distal blocks as the consequence of a non-viable His–Purkinje system at the septal level that makes CSP ineffective. However, it should be noted that in the case of a septal scar located at a basal level only, posterior fascicular pacing can be attempted by targeting the mid and posterior septum, as previously reported by Ponnusamy SS et al. [38]. Moreover, the location of the conduction block at the His–Purkinje level is not completely predictable by the surface QRS morphology, as also recently confirmed in a study by Upadhyay GA et al. [25], where the traditional 12-lead ECG criteria for the LBBB pattern [39] were insufficient for predicting the response to His bundle pacing alone. Therefore, the site of the conduction block can be assessed precisely by using intracardiac data only, and this evaluation should be performed intraoperatively in order to choose between His bundle pacing or LBBAP. Finally, in the presence of distal His–Purkinje disease (e.g., distal bundle branch block or Purkinje network disease), an optimized CRT approach, such as HOT-CRT or LOT-CRT, should be attempted to achieve a greater electrical resynchronization that could not be provided by CSP alone in these specific conditions.

### 2.3. Evidence Gaps and Practical Considerations

Long-term lead performance, lead extractability, and impact on the tricuspid valve function represent the main concerns related to the CSP approach and the data available so far are too scarce to draw conclusions on these matters.

In particular, the impact of septal kinetics on lead durability and therefore on the evolution of the electrical parameters over time in the case of LBBAP is still not fully defined, although lumenless pacing leads could be less affected compared to stylet-driven pacing leads because of the smaller lead body and the high tensile strength.

Data on lead extractability are limited to single-center experiences and case reports [40,41,42,43]. In a series of 30 patients with chronically implanted lumenless His bundle leads (mean dwelling time 25 ± 18 months), the success rate of the extraction procedure was >95%, and no procedure-related complications were observed. In most cases, the leads were extracted by using simple traction, whereas mechanical extraction tools were required only in a few cases [40]. Regarding the LBBP lead, the case reports published so far showed the feasibility of the extraction procedure, describing that lumenless leads implanted in septal position up to 3 years before were removed intact by gentle traction without complications [41,42,43].

Finally, preliminary observations in LBBP patients documented a correlation between the deterioration of the tricuspid valve regurgitation and the distance between the lead-implanted site and the tricuspid valve annulus [44]. This highlights the importance of refining the implant techniques to minimize the interactions of the lead with the septal tricuspid leaflet and the subvalvular apparatus, ideally integrating imaging modalities like intracardiac echocardiography to guide lead placement.

Delivering CSP could be a challenge for patients presenting with specific myocardial diseases such as hypertrophic cardiomyopathy (HCM). The few case reports available so far showed the feasibility of LBBP in HCM patients despite the technical issues related to the amount of septal fibrosis potentially affecting the lead penetration and the pacing threshold values [45,46]. However, more evidence on long-term efficacy and safety is required before CSP can be validated as a standard approach in such complex conditions.

CSP has grown exponentially in clinical practice over the last few years. Additionally, device manufacturers have rapidly developed dedicated implanting tools to reach the target pacing areas more easily and improve the implant success rate. However, device algorithms specifically designed for CRT delivery in CSP settings are still missing. In particular, algorithms able to test and adjust pacing threshold and sensitivity (e.g., for the His bundle pacing lead) as well as adapt atrioventricular conduction intervals to allow fusion pacing in specific settings (e.g., in the case of selective LBBP to avoid a delayed right ventricular activation) [47,48] would be desirable in the near future.

## 3. Future Directions for CRT Delivery

The individualization of CRT modalities based on the patient’s clinical characteristics will hopefully become more and more the adopted strategy in the future in order to maximize the clinical response to CRT and potentially reduce healthcare costs. Recently, the *MADURAI LBBP study* [49] has shown that in patients with non-ischaemic cardiomyopathy, LBBB, and <10% of scar burden at cardiac magnetic resonance, LBBP was associated with a significant improvement in LVEF compared to patients with scar burden > 10%. Furthermore, in patients with low scar burden, no major ventricular arrhythmic events were reported during a mean follow-up of 21 ± 12 months. This preliminary evidence supports the concept that selected heart failure patients can be treated safely with CRT only without defibrillation therapy, potentially reducing the costs related to the implantation of unnecessary devices. However, the development of dedicated tools to help match patient profiles with optimal therapeutic strategies is essential. In this sense, the contribution of artificial intelligence could be meaningful because of the heterogeneity of electrical patterns and myocardial diseases encountered in heart failure patients.

Finally, the miniaturization of technologies to deliver CSP as leadless LBBAP is under development, and more data on safety and feasibility will probably be available over the next few years [50].

## 4. Conclusions

CSP, especially as LBBAP, has progressively gained support as an alternative to conventional BiV pacing to deliver CRT in heart failure patients. However, the lack of data from large randomized studies discourages adopting this approach routinely in clinical practice. Furthermore, conventional epicardial stimulation by a coronary sinus lead still maintains a role, alone or in addition to His bundle pacing or to LBBAP, in specific conditions where CSP seems to be ineffective in restoring electrical resynchronization. Therefore, an individualization of the implant strategy according to the patient’s characteristics appears to be the approach to be adopted in the near future to treat candidates to CRT, aiming to optimize the clinical benefits of this technology.

## Figures and Tables

**Table 1:** Results from the largest series evaluating the feasibility and clinical outcomes of CSP techniques for CRT delivery.

	Authors	Year	Study Design	Total *n*. of pts	Study pts	Intervention	Follow-Up (Months)	Outcomes
**His-CRT**	Upadhyay GA et al. [5]	2019	RCT	41	QRS > 120 ms, LVEF ≤ 35%, NYHA II–IV	His-CRT vs. BiV-CRT	12.2	Greater QRS narrowing in His-CRT pts vs. BiV-CRT pts
	Vinther M et al. [22]	2021	RCT	50	LBBB, LVEF ≤ 35%,	His-CRT vs. BiV-CRT	6	Similar clinical and physical improvement in both groups
**HOT-CRT**	Vijayaraman et al. [8]	2019	Prospective, observational	27	LBBB or IVCD, QRS ≥140 ms, LVEF ≤ 35%, NYHA III–IV	Feasibility study	14 ± 10	Feasibility criteria met Greater QRS narrowing in HOT-CRT vs. BiV or His pacing Significant increase in LVEF and NYHA compared to baseline
**LBBAP-CRT**	Vijayaraman et al. [6]	2021	Retrospective, observational	325	LVEF < 50%, CRT or pacing indications	Feasibility study	6 ± 5	Feasibility and safety criteria met Significant QRS narrowing Significant increase in LVEF and NYHA compared to baseline
	Vijayaraman et al. [7]	2022	Retrospective, observational	121	RBBB, LVEF < 50%, CRT or pacing indications	Feasibility study	13 ± 8	Feasibility criteria met Significant QRS narrowing Significant increase in LVEF and NYHA compared to baseline
**LOT-CRT**	Jastrzębski et al. [9]	2022	Prospective, observational	112	CRT indications or non-response to CRT	Feasibility study	7.8 ± 2.3	Feasibility and safety criteria met Greater QRS narrowing in LOT-CRT vs. BiV or LBBAP Significant increase in LVEF and NYHA and a significant reduction in LVEDV and NT-proBNP compared to baseline

Abbreviations: BiV: biventricular; His-CRT: His-cardiac resynchronization therapy; HOT-CRT: His optimized-cardiac resynchronization therapy; IVCD: intraventricular conduction delay; LBBAP-CRT: left bundle branch area pacing-cardiac resynchronization therapy; LBBB: left bundle branch block; LVEF: left ventricular ejection fraction; LOT-CRT: left bundle branch area pacing optimized-cardiac resynchronization therapy; NYHA: New York Heart Association functional class; LVEDV: left ventricular end-diastolic volume; NT-proBNP: N-terminal prohormone of brain natriuretic peptide; pts: patients; RBBB: right bundle branch block; RCT: randomized controlled trial.

## Data Availability

Not applicable.
